# Fragile X Syndrome and *FMR1* premutation: results from a survey on associated conditions and treatment priorities in Italy

**DOI:** 10.1186/s13023-024-03272-0

**Published:** 2024-07-12

**Authors:** Federica Alice Maria Montanaro, Paolo Alfieri, Cristina Caciolo, Alessia Brunetti, Alessandra Airoldi, Anna de Florio, Luigi Tinella, Andrea Bosco, Stefano Vicari

**Affiliations:** 1 Child & Adolescent Neuropsychiatry Unit, Bambino Gesù Children’s Hospital, IRCCS, Rome, 00165 Italy; 2 Department of Education, Psychology, Communication, University of Bari Aldo Moro, Bari, 70122 Italy; 3 Associazione Italiana Sindrome X Fragile, Piazza Lima 1, Milan, 20124 Italy; 4 Department of Life Sciences and Public Health, Università Cattolica del Sacro Cuore, Rome, 00168 Italy

**Keywords:** *FMR1* gene, Developmental disorders, Fragile X syndrome, *FMR1* premutation, Voice of the patient, Cognitive phenotype, Intellectual disability

## Abstract

**Background and objectives:**

Fragile X Syndrome (FXS) is the most common cause of inherited intellectual disability, caused by CGG-repeat expansions (> 200) in the *FMR1* gene leading to lack of expression. Espansion between 55 and 200 triplets fall within the premutation range (PM) and can lead to different clinical conditions, including fragile X- primary ovarian insufficiency (FXPOI), fragile X-associated neuropsychiatric disorders (FXAND) and fragile X-associated tremor/ataxia syndrome (FXTAS). Although there is not a current cure for FXS and for the Fragile X-PM associated conditions (FXPAC), timely diagnosis as well as the implementation of treatment strategies, psychoeducation and behavioral intervention may improve the quality of life (QoL) of people with FXS or FXPAC. With the aim to investigate the main areas of concerns and the priorities of treatment in these populations, the Italian National Fragile X Association in collaboration with Bambino Gesù Children’s Hospital, conducted a survey among Italian participants.

**Method:**

Here, we present a survey based on the previous study that Weber and colleagues conducted in 2019 and that aimed to investigate the main symptoms and challenges in American individuals with FXS. The survey has been translated into Italian language to explore FXS needs of treatment also among Italian individuals affected by FXS, family members, caretakers, and professionals. Furthermore, we added a section designated only to people with PM, to investigate the main symptoms, daily living challenges and treatment priorities.

**Results:**

Anxiety, challenging behaviors, language difficulties and learning disabilities were considered the major areas of concern in FXS, while PM was reported as strongly associated to cognitive problems, social anxiety, and overthinking. Anxiety was reported as a treatment priority in both FXS and PM.

**Conclusion:**

FXS and PM can be associated with a range of cognitive, affective, and physical health complications. Taking a patient-first perspective may help clinicians to better characterize the cognitive-behavioral phenotype associated to these conditions, and eventually to implement tailored therapeutic approaches.

**Supplementary Information:**

The online version contains supplementary material available at 10.1186/s13023-024-03272-0.

## Introduction

Fragile X Syndrome (FXS) is a rare genetic syndrome caused by the expansion of the CGG repeat in the 5’ untranslated region of the *FMR1* (Fragile X Messenger Ribonucleoprotein 1) gene located on the X chromosome [7]. This expansion results in the methylation of the *FMR1* promoter, transcriptional silencing of the gene and consequently in the reduction or absence of the FMR1 protein [37]. FXS represents the leading known inherited cause of intellectual disability (ID) and the most common cause of autism spectrum disorder (ASD) due to a single-gene mutation [12]. The average IQ (intellectual quotient) in men is 40–50, while women are usually less affected as they present a second unaffected copy of the X chromosome. A characteristic cognitive-behavioral phenotype is also recognisable: the 80% of males and the 40% of females meet criteria for attention-deficit hyperactivity disorder (ADHD), while anxiety is present in the 58%-86% of individuals [9]; obsessive–compulsive disorder has been also reported [9]. Other behavioural features include hypersensitivity to stimulation, social gaze avoidance and aggression; language deficits both in comprehension and in production are also common [23]. Furthermore, children and adults with FXS exhibit impairment in different domains of adaptive functioning, which can be considered as the ability to cope with everyday environmental demands and to live independently [12, 9].

FXS represents the most investigated X-linked condition, however there is a spectrum of clinical manifestations associated with *FMR1* premutation (PM) that occurs when CGG trinucleotide repeats between 55 and 200 times. PM is common in general population [12] and its pathophysiology is due to higher than normal levels of *FMR1* mRNA in the PM range, which leads to “RNA toxicity” that results in different clinical conditions falling under the umbrella term of Fragile X Premutation Associated Conditions (FXPAC) [9]. FXPAC include different disorders, such as Fragile X-associated Tremor/Ataxia Syndrome (FXTAS) and Fragile X-associated Primary Ovarian Insufficiency (FXPOI) [12, 8, 12, 9]. Furthermore, research has shown that people with PM may experience some mental and physical health symptoms that are more frequent and significantly different from FXTAS and FXPOI and that usually occur at an earlier stage that the last two. For this symptomatology in 2018 it has been proposed the term Fragile X-associated neuropsychiatric disorders (FXAND), which are thought to affect at least half of PM carriers [12]. This umbrella term includes different conditions, such as anxiety, depression, ADHD, and socio-pragmatic difficulties ([i.e., [9, 9]). Connective tissue problems, hypertension, or autoimmune problems, chronique fatigue and fibromyalgia, which are not covered by FXTAS, FXPOI and FXAND are also more frequently diagnosed in people with PM if compared to general population [9]. Furthermore, recent advances in the PM characterization over the lifespan have shown that neuropsychiatric problems start in early years, even though the PM phenotyping in childhood is still an open challenge [27].

Cognitive deficits, together with physical, behavioural, and psychological problems interfere with development and with different daily living skills, such as making friendships, finding a job or participating to society activities, therefore contributing to the adaptive functioning impairment in adult life [23, 23]. Studies using heterogeneous samples of adults with ID have shown that co-occurring conditions may worsen the disability and the quality of life (QoL) of those individuals [42]. To date, there has been qualitative research aimed to explore the impact of co-occurring mental issues on independence and the major concerns in FXS and associated conditions. For instance, Bailey and colleagues conducted a national family survey [3, 4] in order to understand the needs of families who had at least one child with FXS or PM. The authors found that most adults with FXS mastered many daily-life skills, with women functioning averagely better than men. At the same time, there were different skills more difficult to be attained, such as engaging in conversations and speaking clearly. Furthermore, several characteristics, including overall thinking, reasoning, and learning ability, adaptability, ability to pay attention, and total number of co-occurring conditions, were predictive of functional skills for both males and females with FXS. Additionally, the survey showed that one third of people with PM had been diagnosed or treated for developmental delay, with a strong correlation between number of co-occurring conditions and QoL not only in FXS but also in PM. Later, Hartley et al. [12], based on the data collected by Bailey et al. [4], described the adult lives of 328 individuals with FXS, evidencing that the levels of functional skills were the strongest predictors of independence in adulthood and that interpersonal skills as well as co-occurring psychological issues should be a target intervention as well as ID. Later, Cross et al., [12] conducted a study in which they asked to 612 caregivers of males with FXS to choose the best treatment priorities. Controlling behaviour and caring for oneself resulted the most-selected choices, independent of the age of the individual with FXS. This survey was helpful for clinicians in designing targeted interventions; however, this study was directed only to males with FXS, therefore not resulting equally informative for the population of females with FXS and of people with PM. Afterwards, with the aim to expand understanding of the needs of individuals with FXS by taking a patient-first perspective, Weber et al. [9] conducted an online survey completed by 467 American respondents, including family members/caretakers, but also professionals and individuals with FXS. The survey was designed to investigate the main symptoms, daily living challenges, family impact and treatment priorities. Respondents indicated that the main areas of concern were anxiety, behavioural problems, and learning difficulties. Anxiety was also described as a top treatment priority by all the categories of respondents, including people with FXS. Although there is growing data from surveys on population with FXS, to date much of research on FXS and related disorders has been primarily conducted in American communities, potentially limiting the generalizability of findings to other countries, where socio-cultural factors may lead to different outcomes. To our knowledge, Italian surveys on FXS and related conditions have never been conducted. Trying to address this gap, we translated the survey developed by Weber et al. [9] into Italian language in order to collect information more representative of Italian culture. Specifically, our primary objectives were twofold:

1. To investigate main symptomatology and treatment needs of Italian individuals affected by FXS, as perceived by the individuals themselves, their family members, caregivers, and professionals within the Italian context.
2. To investigate main symptomatology and treatment needs of Italian PM carriers, by adding a section specifically designated for them. Our objective was to amplify the voices of a population often neglected in comparison to FXS, despite being associated with distinct clinical manifestations.

Both for FXS and PM, results could be beneficial for clinicians seeking to create treatments that are truly tailored to meet the specific needs of their patients in their environmental context.

## Methods

### The Italian survey

The Italian survey is the result of the translation of the American version that Weber and colleagues created in collaboration with the National Fragile X Foundation [9]. The original survey included forced-choice, ranking and open-ended questions about the following information: (1) Respondent category (individual with FXS, family member/caretaker, professional); (2) age of the person with FXS; (3) problematic symptoms; (4) major concerns; (5) daily living skills most affected; (6) family impact of symptomatology; (7) the most three favourite aspects about the person with FXS (or about yourself); (8) top three treatment priorities. The Italian survey aimed to investigate the same areas with the same number of questions, but with two main differences: 1) In the question 3–4, we added other items to the ones already present in the original survey; 2) we did not use ranking questions because the Italian respondents found it difficult to assigning a ranking score; for this reason, in the survey we used 5 points-Likert type questions allowing respondents to assign a score from 1(most impacting) to 5 (least impacting) to each answer option.

Additionally, we added a section designated only for PM carriers, aiming to investigate the following areas: (1) gender; (2) age; (3) major concerns; (4) daily living skills most affected; (5) family impact of symptomatology; (6) any diagnosis received (FXTAS, FXPOI, FXAND, other); (7) top three treatment priorities. This section that was fillable only by persons with PM of both genders, used the same compilation method of the section on FXS. People (parents, caretakers or professionals) associated with more than one person with FXS, could complete as many surveys as the number of affected people. Furthermore, people with PM could fill either the entire survey or only the section designated for PM. A mother with PM (i.e., a mother of more than one child with FXS) who completed more than one survey, filled the section for PM only once. The Italian survey can be consulted in the Appendix A.

### Procedure

The creation of the survey as well as the access to the Excel file was solely managed by the Italian Fragile X Syndrome Association. Link to the survey (via Google Forms) was emailed to all contacts registered to receive emails from the Italian Fragile X Syndrome Association or was disseminated through the Association's main social channels (Facebook, Instagram). Respondents anonymously answered to the survey and the consent to publish data was given by clicking on a checkbox.

### Sample

One hundred and thirty-eight people completed the survey about FXS, including 4 people with FXS (2 females and 2 males), 62 mothers with PM, 7 males with PM, 5 females with PM (without children), 36 family members/caregivers, and 24 professionals (all teachers). Finally, 96 individuals (87 females and 9 males) answered to the section on PM. Unfortunately, other sociodemographic information of the respondents could not be collected due to the privacy policy of the Italian Fragile X Syndrome Association.

### Analysis

In the section on FXS we described data separately for category of respondents (self, males with PM, females with PM, family members/caretakers; mothers with PM; professionals), while in the section on PM we qualitatively analysed answers distinguishing by respondents’ gender (male *vs* female) and age (0–21 years; 22–45 years; 46–65 yeas; 66 years and over). More specifically, data were analysed as follows:

1. Forced-choice questions: for each item, we calculated percentages of responses within groups by category, age, and sex.
2. Likert type questions: to compare ranked answers, we calculated a weighted mean rank score. In detail, we assigned the highest weight (5) to the items ranked first, giving a dropping weight as ranking place decreased. For each item, the score was the sum of all the weighted values, divided by the number of respondents for each subgroup. In the analysis we also included data from participants who did not rank all the items (e.g., only assigning ranks of 1 and 2 out of 5 and leaving all others blank). It must be specified that, as the sample size was really different among sub-groups we could not directly compare the different mean-scores. Therefore, we decided to conduct a qualitative analysis, identifying for each question the top 3 ranked items by subgroups of respondents.
3. Open ended questions: here, data were first open coded for themes; thereafter a keyword was associated to each theme. Then, for each item we calculated percentages of responses distinguishing between sub-groups.

Given that each question comprised numerous items, here we present only the top three answers for each category of respondents.

## Results

### The section on FXS

#### Charateristics of respondents

**Fig. 1:**
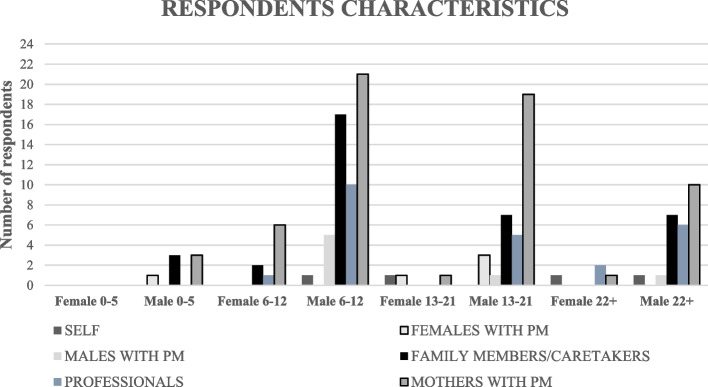
Survey respondents’ characteristics. FXS = Fragile X Syndrome; PM = FMR1 Premutation

Figure 1 shows demographic characteristics of respondents. Most of reporters responded for males with FXS aged 6–12 (54/138 = 39%). It should be noted that no one responded for females aged 0–5. Most respondents were mothers with PM (62/138 = 45%).

#### FXS- Problematic symptoms

Respondents completed the following question: “Assign a score from 1 to 5 to the characteristics below based on how much you think they impact on the life of the person with FXS”. Table 1 shows the mean weighted top 3 rank scores for each subgroup of respondents.

**Table 1:** FXS- Problematic symptoms

	**Self**	**Males with PM**	**Females with PM**	**Mothers with PM**	**Family Members/Caretakers**	**Professionals**
1st	ANX (4)	HYPER (4.7)	PRAGMATICS (4.3)	ID (4)	ANX (4)	EXPRESSIVE (4)
2nd	ID (3.3)	MOTOR ST/ANX (4.2)	EXPRESSIVE/ MOTOR ST (4)	SCHOOL/ WORK (3.9)	SCHOOL/WORK (3.8)	ID/ MOTOR ST (3.9)
3rd	VISUAL (3)	ATTENTION (3.9)	ANX (3.7)	EXPRESSIVE (3.8)	ID (3.7)	PRAGMATICS (3.7)

*ANX* Anxiety, *ATTENTION* Short attention span, *EXPRESSIVE* Expressive language, *HYPER* Hyperactivity, *ID* Learning or intellectual disability, *MOTOR ST* Motor stereotypes, *PRAGMATICS* Difficulties in social-relational autonomies, *SCHOOL/WORK* Difficulties in work and/or schooling, *VISUAL* Visual information processing difficulties

Values in the round brackets = mean rank scores

Out of potential symptoms listed, “anxiety” represents one of the top three symptoms according to almost all the subgroups and the highest mean rank score for both people with FXS and family members. Interestingly, mothers with PM did not consider anxiety a top problematic issue for their children. Other high rated scores were expressive language (spoken language) and ID, with the latter being considered the symptom having the greatest impact by the group of mothers. Finally, the item “school/work difficulties” was the second highest rated score both by family members and by mothers.

#### FXS- Primary concerns

Respondents answered the following question: “Assign each of the following areas a score from 1 to 3 based on the extent to which you think it affects the daily life of the person with FXS”. Table 2 highlights rank mean scores for each subcategory of compilers.

**Table 2:** FXS—Primary concerns

	**Self**	**Males with PM**	**Females with PM**	**Mothers with PM**	**Family Members/Caretakers**	**Professionals**
1st	LANG/INTEL (2.2)	BEHAV (2.5)	LANG/INTEL (2)	LANG (2.5)	BEHAV (2.3)	LANG (2.5)
2nd	PHYS (2)	LANG (2.3)	BEHAV (1.8)	BEHAV (2.4)	LANG (2.2)	BEHAV (2.3)
3rd	BEHAV (1.8)	PHYS (2)	PHYS (1.6)	INTEL (2.2)	INTEL (1.9)	INTEL (2.1)

*BEHAV* Behaviour, *INTEL* Intelligence, *LANG* Language, *PHYS* Physical

Values in the round brackets = mean rank scores

Language and behaviour were the averagely highest rated concerns by all categories of respondents, while physical abilities were never placed at the 1st position by any of subgroups.

#### FXS- Daily living skills most affected

Participants responded to the question: “Assign a score from 1 to 5 to the areas of daily life listed below based on how much you think they are affected by in the person with FXS”. Table 3 exhibits the top three areas with the highest mean scores distinguished by groups of respondents. To note, “ability to learn academic skills” is the highest rated score in almost all the groups’ opinion. Highly voted are also “ability to communicate” and “ability to attend events where there are a lot of people/noise”. Interestingly, “ability to take care of oneself ” is one of the top three voted items only by professionals.

**Table 3:** FXS—Daily living skills most affected

	**Self**	**Male swith PM**	**Females with PM**	**Mothers with PM**	**Family Members/Caretakers**	**Professionals**
1st	ACADEMIC (4.3)	ALONE (4.9)	ALONE/SCHOOL(3.3)	ACADEMIC (4.4)	ACADEMIC (4.5)	ACADEMIC (4.1)
2nd	ANGER (3.3)	ACADEMIC (4.7)	COMM/ACADEMIC/HOLIDAYS/ WORK (3)	PEERS/ INDEPENDENCE (4)	PEERS (4.2)	COMM/ALONE (4)
3rd	FRIENDSHIP (3)	PEOPLE-NOISE (4.4)	PEOPLE NOISE (2.8)	RELATIONSHIP (3.8)	COMM (4.1)	ANGER/SELF CARE (3.8)

*ACADEMIC* Learn academic skills, *ALONE* Be left alone, *ANGER* Control behavioural outbursts, *COMM* Speak/communicate, *FRIENDSHIP* Ability to make and maintain friends, *FRIENDSHIP* Maintain friends, *HOLIDAYS* Willingness to travel/ go on vacation, *INDEPENDENCE* Live independently, *PEERS* Be like people of the same age, *PEOPLE-NOISE* Events where there are people/noise, *RELATIONSHIPS* Start a relationship, *SCHOOL* Attend school, *SELF-CARE* Take care of self-care skills, *WORK* Find/maintain a job

Values in the round brackets = mean rank scores

#### FXS- Family impact

Respondents answered the question: “Score each of the following aspects from 1 to 5 according to how challenging you think it is to live with FXS”. As Table 4 depicts, “worry for the future” was ranked as the main challenge by both people with FXS and mothers with PM. Furthermore, out of twenty potential options, “planning” and “always thinking” were considered one of the most challenging aspects by all categories of respondents excluding professionals. Only professionals considered “food” one of the most impacting feature of FXS.

**Table 4:** FXS—Family impact

	**Self**	**Males with PM**	**Females with PM**	**Mothers with PM**	**Family Members/Caretakers**	**Professionals**
1st	FUTURE (5)	PLANNING (4.5)	MEDICATION/SLEEP (4)	FUTURE (4.2)	PLANNING (4.3)	FOOD (4.4)
2nd	THINKING (4)	THINKING/RELAX (4.4)	PLANNING (3.4)	PLANNING/ THINKING (4.1)	FUTURE (4.2)	MEDICATION (4)
3rd	EXTRATIME/NEEDS (3)	EXTRATIME/NEEDS/ SUPERVISION (4.2)	FUTURE/DOCTORS/ FRIENDS (3.3)	RELAX (3.9)	THINKING/ SUPERVISION (4)	ROUTINE (3.9)

*DOCTORS* Doctor appointments, *EXTRA-TIME* Extra time it takes to do everything, *FOOD* Always hungry, *FRIENDS* Activities with friends, *FUTURE* Worry about the future, *MEDICATION* Getting prescriptions, *MEDICATION* Getting prescriptions/not running out/ making changes, *NEEDS* Person is unable to tell you what he/she wants/needs, *PLANNING* Checking in/setting up daily programming—school, work, etc., *RELAX* Finding respite, *ROUTINE* Need to make sure that everything is set for the day”, *SLEEP* Sleeping, *SUPERVISION* Need for constant supervision, *THINKING* Always thinking – how are things going

Values in the round brackets = mean rank scores

#### FXS—Treatment priorities

**Fig. 2:**
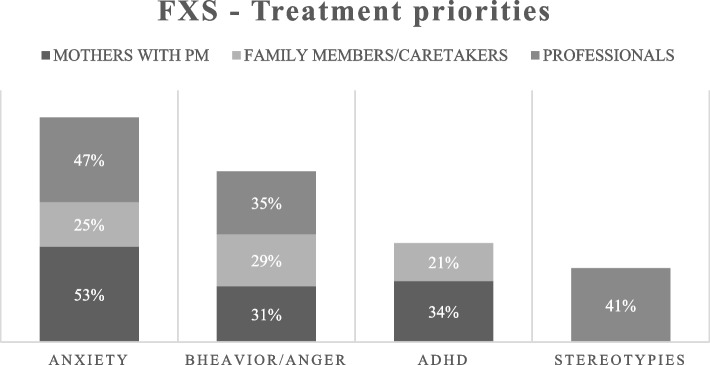
Treatment priorities. FXS = Fragile X Syndrome. PM = premutation. Values in the squares = percentages of subgroups’ respondents

Respondents answered the following question: “What are the top three aspects of Fragile X Syndrome that you would like to see a treatment address?” We coded answers for themes, and we depicted their frequencies among mothers, family members/caretakers and professionals (see Fig. 2). This question was answered by 47 mothers, 31 family members/caretakers and 17 professionals. Moreover, three family members expressed their lack of interest in any form of treatment, while 11 out of 47 mothers clarified that their responses pertained specifically to behavioural interventions and not pharmacological treatments. Here, we did not include answers from people with FXS, males and females with PM because of the low numerousness of compilers. In general, individuals with FXS asked help for anxiety and emotion management, while individuals with PM pointed out the necessity to treat attention/hyperactivity together with anxiety.

### The section on PM

#### Respondent characteristics

**Fig. 3:**
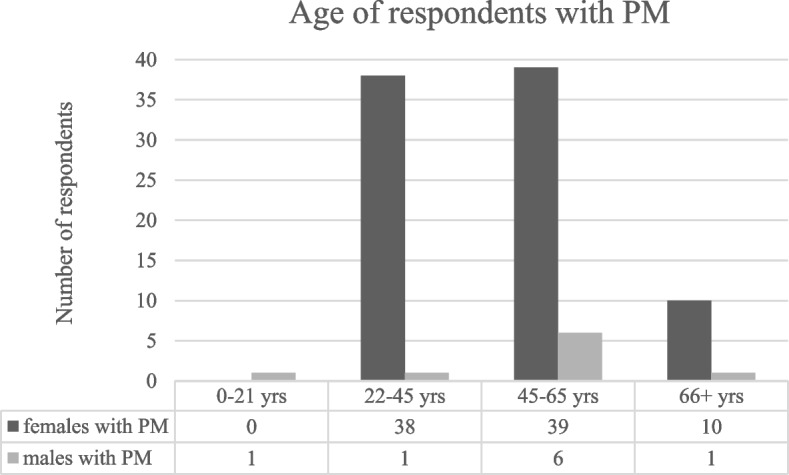
Characteristics of respondents with PM (FMR1 premutation)

The section on PM was compiled by 96 individuals, of which 87 females (including the 62 mothers with PM that also filled the section on FXS) and 9 males. As Fig. 3 shows, most respondents were females aged 45–65, followed by females aged 22–45 and 66 years and over.

#### PM- Problematic symptoms

Respondents with PM answered the following question: “Assign a score from 1 to 5 to the characteristics below based on how much you think they impact on your life”. Table 5 depicts the highest weighted mean scores for females with PM. From the survey, it emerges that memory (both short and long term) problems, social anxiety and attention deficits were the main problematic symptoms for all the age-groups of females with PM.

**Table 5:** Females with PM—Problematic symptoms

	**22–45 yrs**	**46–65 yrs**	**66 + yrs**
1st	LTM (2.8)	STM (2.1)	STM/ATT (3.1)
2nd	SANX (2.6)	LTM (1.8)	LTM (2.7)
3rd	STM (2.5)	SANX (1.5)	SANX (2.6)

*ATT* Attention deficits, *LTM* Long-term memory, *SANX* Social anxiety, *STM* Short-term memory

Values in the round brackets = mean rank scores

Considering males with PM, only 9 individuals responded to survey, of whom three did not answer to this question. The only individual aged 0–21 and the one falling in the last range of age agreed that short-term memory deficits were main symptom. According to males aged 45–66 years, attention deficits followed by perseveration and pragmatic problems were considered the major concerns.

#### PM- Daily living skills most affected

Compilers completed the question: “Assign a score from 1 to 5 to the areas of daily life listed below based on how much you think they affect your life”. Taking into account females with PM, out of 14 possible areas, the “ability to attend events where there are a lot of people/noise” was placed at the 1st position by all subgroups of age; other highly rated items were the “ability to learn academic skills”, “ability to maintain friends” and the “ability to control behavioural outbursts” were in the top three mean rank scores for all the age-groups (Table 6).

**Table 6:** Females with PM- Daily living skills most affected

	**22–45 yrs**	**46–65 yrs**	**66 + yrs**
1st	PEOPLE/NOISE (3)	PEOPLE/NOISE (2.9)	PEOPLE/NOISE (3.4)
2nd	ACADEMIC (2.4)	ANGER (1.9)	ANGER (2)
3rd	FRIENDSHIP (2.3)	ACADEMIC (1.7)	ACADEMIC/ FRIENDSHIP (1.7)

*ACADEMIC* Learn academic skills, *ANGER* Control behavioural outbursts, *FRIENDSHIP* Maintain friends, *PEOPLE/NOISE* Events where there are people/noise

Values in the round brackets = mean rank scores

Regarding males with PM, both the youngest and the oldest male reported that learning academic skills was the most affected ability, followed in the first case by the ability to take care of himself and in the latter by the ability to be left alone. The only individual in the age range 22–45 years scored only “ability to speak/communicate” and “ability to attend school” assigning them the highest value. The remaining group of males (46–65 years) averagely agreed that academic skills were the most affected, followed by the ability to be left alone.

#### PM- Family impact

Respondents scored the question: “Score each of the following aspects from 1 to 5 according to how challenging you think it is to live with PM". Table 7 shows the top three items selected by females with PM. Here, there is full agreement between groups to consider “worry about the future”, “the ability to relax” and “the over-thinking” the main challenging aspects. To note, “extra time needed to do everything” is in the top three items only for the oldest age range of females, achieving also the highest mean score.

**Table 7:** Females with PM—Family impact

	**22–45 yrs**	**46–65 yrs**	**66 + yrs**
1st	RELAX (4)	THINKING (3.6)	EXTRA-TIME (4.9)
2nd	THINKING/FUTURE (3.9)	FUTURE (3.3)	FUTURE (4.3)
3rd	EXTRA COST (3.7)	RELAX (3)	RELAX (3.6)

*EXTRA- COSTS* Therapies, medications, clothing, glasses, laundry, *EXTRA TIME* Extra time it takes to do everything, *FUTURE* Worry about the future, *RELAX* Finding respite, *THINKING* Always thinking – how are things going

Values in the round brackets = mean rank scores

In the category of males, only the youngest men and three men aged 46–65 years answered to this question. The first one scored only three items (“thinking”, “future” and “finding/attending doctors’ appointments”) assigning to all of them the highest grade. The other men averagely considered the most challenging aspects “future” and “over-thinking”.

#### PM—Comorbidities and treatment priorities

**Fig. 4:**
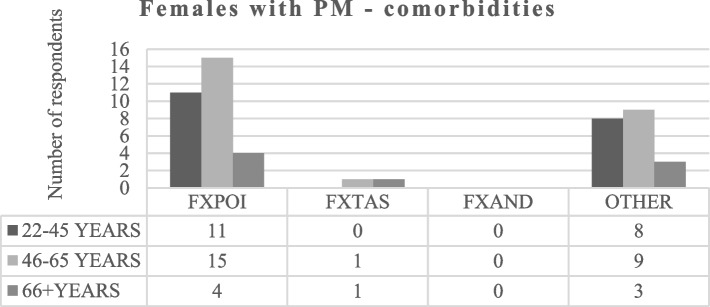
Comorbidities in females with PM. FXPOI = Fragile X-associated Primary Ovarian Insufficiency. FXTAS = Fragile X-associated Tremor/Ataxia Syndrome. FXAND = Fragile X-associated neuropsychiatric disorders

**Fig. 5:**
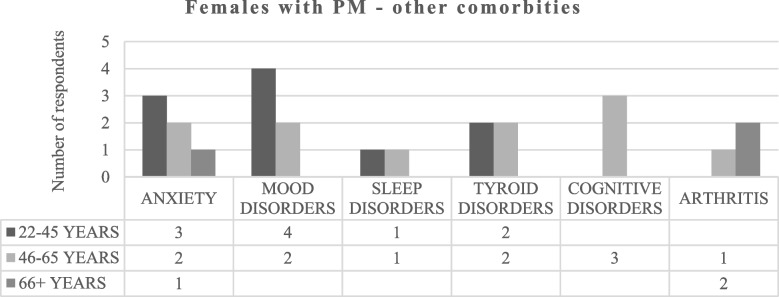
Other comorbidities in females with PM

Individuals with PM were asked to respond to the following questions: “Specify if you suffer of FXPOI, FXTAS, FXAND, other (specify)” and “What are the top three aspects of PM that you would like to see a treatment address?” None of the males answered to these questions. Taking into account females with PM, the first question was answered by 52 respondents (19 women aged 22–45, 25 women aged 46–65 and 8 women aged 66 years and over). Figure 4 shows that FXPOI is the most frequent diagnosis among respondents, and no woman indicated presenting FXAND. However, when asked to specify if they suffered from other disorders, four women affirmed having anxiety, six women reported mood disorders, two reported sleep disorders, and three reported cognitive disorders, all of which fall under the umbrella term of FXAND (Fig. 5).

**Fig. 6:**
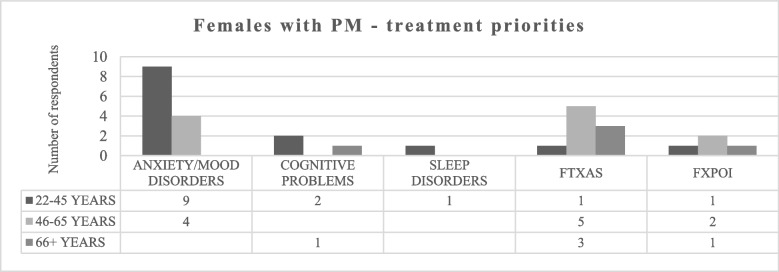
Treatment priorities for females with PM. FXPOI = Fragile X-associated Primary Ovarian Insufficiency. FXTAS = Fragile X-associated Tremor/Ataxia Syndrome

Finally, 30 females responded to the questions about treatment priorities. As Fig. 6 underlines, treatment for anxiety/mood disorders was most popular one.

## Discussion

Aim of the present work was to expand the survey of Weber and colleagues [9], exploring the main areas of concern and treatment priorities among Italian individuals with FXS. Furthermore, we added a new section specifically designated for people with PM in order to investigate the main symptoms, associated clinical disorders and treatment needs also in this condition.

Taking into account the section on FXS, while our initial goal was to understand if different subcategories of respondents would have provided statistically different answers, the inhomogeneous numerousness of sub-groups did not allow us to perform comparative analyses. Nevertheless, we conducted qualitative analyses highlighting the most frequently selected items by different subgroups of respondents. Before discussing these results, it must be said that, as in Weber and coll. [9], there were very few individuals with FXS answering to the survey, which could be due to the difficulty for people with ID in responding to a questionnaire with a lot of questions and options. Furthermore, most of respondents compiled the questionnaire for males with FXS aged 6–12. Therefore, it is essential to interpret findings cautiously and avoid generalizing them to the entire Fragile X population.

Additionally, despite our decision to translate Weber's survey into Italian language, it's important to note that we couldn't directly juxtapose our results with those of Weber and colleagues [9]. This discrepancy arose because our respondents encountered challenges in ranking only five items per question on a scale from 1 to 5. Consequently, we had to employ a different methodology from the one utilized by the authors; indeed, we asked to the compilers to assign to each item a score from 1 to 5, subsequently selecting the highest mean scores for each question and presenting them in the paper. Moreover, in contrast to Weber and colleagues [9], who divided respondents into only three categories (self, family members/caretakers, professionals), we made a more detailed distinction. Specifically, we separated the responses of mothers with PM from those of other family members with PM, as well as from other relatives without PM. Nevertheless, while a direct comparison was not possible, our results are in some way consistent with the ones of Weber; specifically, anxiety emerged as the most concerning symptom of FXS among individuals with FXS and their family members, ranking within the top three answers across all respondent groups except for mothers with PM. Intriguingly, while mothers with PM identified ID as the primary challenge, anxiety did not averagely rank within their top three concerns. The explanation of this result could be twofold: firstly, one conceivable speculation is that mothers, identifying anxiety within themselves as a significant symptom (Table 5), viewed it as less worrisome in their children; secondly, it is also viable to consider that mothers deemed factors such as IQ, academic skills, and expressive language to be more pressing concerns. This perspective aligns with existing literature highlighting the influential role of IQ, learning capabilities, and speech in adaptive functioning and QoL, both within FXS and broader neurodevelopmental disorders (NDs) [1, 12, 23]. In all instances, mothers with PM agreed with other Italian subgroups of respondents and with the American compilers [9] in considering anxiety a treatment priority.

Expressive language was advised as another significant problematic symptom, which is in line with previous works reporting that language difficulties represent one of the greatest challenges and a primary concern of FXS ([i.e., [2, 12, 9]). This is to be expected considering the strong correlation between expressive language skills of children with FXS and their capacity for independence in adulthood [1]. Therefore, it is in some way surprising that Italian respondents, while pointing out the ability to communicate as one of the daily living skills most affected, did not rate language as a treatment priority. Nonetheless, it's plausible that this result is partially influenced by the extensive utilization of speech therapy in the clinical management of NDs, especially among children aged 0–7 years. Consequently, respondents may have perceived this issue as less urgent in terms of treatment needs.

Our results are also consistent with Bailey [3, 4], since we found that learning disabilities, overall thinking and planning as well as the difficulties in attending events where there are lots of people/noise, were strongly associated to FXS phenotype. Furthermore, in line with Cross and colleagues [12], challenging behaviors were reported as primary concerns, which is consistent with data in literature showing that individuals with FXS exhibit elevated rates of behavioral problems ([i.e., [9]) that are considered to have, together with psychiatric issues, the greatest impact (more than ID) to the QoL of individuals with FXS and their families [12]. Effectively, in accordance with those premises, Italian respondents considered anxiety followed by behavioral issues, a priority for intervention, with 11 out of 47 mothers responding that their answers referred to a behavioral treatment and not to medication. One possible explanation to the higher choice of anxiety as the main focus of treatment, could be that non-pharmacological early interventions like Applied Behavior Analysis (ABA) are commonly recommended and have proven effective in managing challenging behaviors in children with FXS [27, 27]. In contrast, treatments for anxiety, such as cognitive behavioral therapy (CBT), have received less attention despite some initial evidence supporting their efficacy [9]. As a result, there remains a high demand for interventions tailored specifically to addressing anxiety in this population. Furthermore, it must be said that, contrastively to the robust data on the FXS phenotype, to date there are still not systematic guidelines for non-pharmacological treatment in FXS [23], reason for which respondents could consider behavioral interventions as more urgent.

These findings are valuable both for research and clinical purpose; indeed, on one hand they direct toward the development of novel research lines (i.e., to testing novel behavioral interventions) and on the other hand they guide clinicians in identifying treatment goals.

Considering data on FXS section as a whole, it can be concluded that, exactly as in Weber and colleagues [9], Italian respondents considered anxiety, learning abilities and behavior the main concerning problems in people with FXS, with the relevance of each of these areas varying based on the focus of the question and the category of respondents. More specifically, while mothers with PM seem to be more worried about the impact of ID/academic problems in the future of their children with PM, other respondent categories express greater concern about anxiety, behavior and language skills. Interestingly, motor stereotypies were reported as a main symptom from almost all the categories of compilers and a treatment priority from professionals’ perspective. As stereotypies in FXS and more in general in NDs can be interpreted as a manifestation of anxiety [6], it cannot be excluded that the underlying symptomatology for which respondents declared to be worried was the same. In this regard, future diagnostic manuals should aim to provide clearer explanations of anxiety and other psychiatric disorders in individuals with ID. This would enable patients, caregivers, and clinicians to better understand the clinical manifestations associated with NDs.

Considering the PM section, our initial goal was to directly compare responses from females and males. However, due to only 9 males completing the online questionnaire, quantitative analysis was not feasible. Before delving into qualitative findings, it's crucial to highlight that although PM appears to be more prevalent among women [9, 12], this doesn't justify the significant gap between female and male respondents; one explanation could be that women are in general more likely to answer questionnaires/surveys than men [5]; another possible interpretation is that a man with PM does not pass on a full mutation to any of his children, therefore remaining unaware of the condition unless other family members do not present FXS; consequently, it's plausible that various Italian male carriers are still unaware of their diagnosis [12]. The reason for which we decided to include PM in our survey is because the condition has long been considered asymptomatic, with males with PM called nonpenetrant and transmitting males. With time, it has been shown that individuals with PM are at increased risk for several health concerns, often ignored not only by carriers themselves but also by clinicians. Those concerns include FXTAS, FXPOI and other multiple medical conditions [9], but also anxiety, ADHD, social deficits, depression, and cognitive problems that fall under the category of FXAND [9]. Our results are consistent with previous data, indeed females with PM of all ages reported short and long memory problems together with social anxiety as main problematic symptoms. To note, the daily life skills considered to be the most affected by all the women with PM were the inability to attend places where there are a lot of people/noise, a symptom that can also be considered a sign of social anxiety [9] and that has already frequently depicted in women with PM [i.e., [9]). Other main concerns that emerged as consistently problematic were academic problems and difficulties to cope with anger. These results are in some ways in line with the survey of Bailey and colleagues [3, 4] in which learning disabilities and autistic traits were strongly associated with PM and in line with previous studies on the neurobehavioral phenotype associated to PM [23]. Additionally, the worry for the future, the inability to relax and overthinking that represent a core symptomatology in the cognitive model of anxiety and depression [9], were considered by Italian respondents to be the most challenging aspects of PM. This result aligns with the literature showing that anxiety and depression are commonly experienced by people with PM and that this is not due only to raising up a child with FXS [9, 9]. To note, while reporting anxiety as a major concern, none of the compilers declared to suffer of FXAND. This result could be due to a dual interpretation: on one hand, it is possible that Italian compilers are not aware about the meaning of FXAND; on the other hand, we cannot exclude the possibility that they dislike the terminology therefore deciding not to use it in the survey. This second interpretation is supported by previous studies underlying that carries find the term FXAND as stigmatizing ([i.e., [12]) and by the fact that, when asked to specify if they suffered of any comorbidity, Italian respondents did not hide to exhibit anxiety, mood disorders, sleep difficulties and cognitive deficits which all fall under the umbrella term of FXAND. Furthermore, anxiety and mood disorders were considered the top treatment priorities, which confirm that our respondents show insight about their daily life difficulties. This data is important and raises areas for discussion. Indeed, in a future work it should be investigated if carriers are aware that psychiatric in PM are not due only to having a child with ID, but also to specific genetic factors (elevated *FMR1* mRNA, mitochondrial dysfunction and calcium dysregulation underline the pathophysiological changes seen in PM) [27, 27]. Further studies aiming to investigate the effectiveness of behavioral interventions (i.e., psychoeducation) in this population should be pursued, too.

Interestingly, Italian respondents with PM identified memory and attention problems as primary concerns, yet they did not prioritize cognitive intervention. This could be attributed to a lack of awareness about the availability of neuropsychological treatments or, alternatively, the perception that anxiety/mood disorders have a greater impact on daily life when compared to other symptoms. It is also possible that subjective memory complaints may be indicative of anxiety, particularly among younger respondents, and that addressing psychiatric issues could also benefit cognitive deficits. Future research on treatment strategies among carriers is needed to address these important, still unanswered questions.

In summary, Italian data emerging from this survey, totally align with previous findings on PM, suggesting that some people with PM may exhibit health problems that are different from FXTAS and FXPOI and that should receive more attention by clinicians. Genetic screenings are now more accessible than ever before and should maybe be considered from both preventive and early treatment perspectives.

In summary, this work represents the first Italian survey to provide information about FXS and PM phenotyping and treatment priorities assuming a patient/caregiver first perspective. Our results, consistently with the ones of Weber [9], encourage clinicians to carry out cognitive-behavioral and neuropsychological interventions in the treatment of the symptomatology associated with both FXS and PM and researchers to develop novel targeted interventions. This need becomes even more critical given the significant lack of research on psychological interventions for mental health issues in individuals with NDs. The limited understanding of effective therapeutic approaches for this population underscores the urgency for more comprehensive studies and targeted treatment strategies. Addressing this gap is essential for improving the mental health outcomes and overall well-being of individuals with NDs [23].

### Limitations and future directions

The study presents the following main limitation: 1) as the survey was anonymous and conducted online, we did not have the possibility to confirm the correctness of the information provided; on the other hand, the aim of the study was to replicate the work of Weber and colleagues [9] giving voice also to Italian respondents; for this reason, we translated the American survey rather than validating it into Italian language, which could represent another limitation; 2) the structure of the survey, which included different questions and options, could be difficult to be compiled by some people (mostly by the ones with ID or cognitive difficulties); therefore, future surveys should use a design more appropriate and accessible by everyone (i.e., with less options); 3) finally, the sample-size among subcategories was inhomogeneous not allowing us to perform direct comparison. Future research should provide more homogeneous sample-sizes to allow direct comparisons and perform proper statistical analysis.

## Conclusions

In conclusion, despite limitations, these qualitative data provide a reference point for Italian clinicians working with FXS and PM and for future research in this field. From a clinical perspective, these findings offer documentation about families’ needs and clinical priorities, pointing anxiety as a treatment priority for both populations. We hope that these results could be the basis for future systematic research on the efficacy of different interventions for FXS and FXPAC.

### Supplementary Information

Additional file 1.

## Data Availability

The data sets generated and analyzed during the study are not publicly to protect study participant privacy, but they are available from the corresponding author on justified request.

## Abbreviations

ABA: Applied Behavior Analysis
ADHD: Attention-deficit hyperactivity disorder
ASD: Autism spectrum disorder
CBT: Cognitive behavioral therapy
FMR1: Fragile X Messenger Ribonucleoprotein 1 gene
FXAND: Fragile X-associated neuropsychiatric disorders
FXPAC: Fragile X-PM associated conditions
FXPOI: Fragile X- primary ovarian insufficiency
FXS: Fragile X Syndrome
FXTAS: Fragile X-associated tremor/ataxia syndrome
ID: Intellectual disability
IQ: Intellectual quotient
LTM: Long-term memory
NDs: Neurodevelopmental disorders
PM: Premutation
QoL: Quality of life
SANX: Social anxiety
STM: Short-term memory
