# Supplementary material for: Fragile X Syndrome and FMR1 premutation: results from a survey on associated conditions and treatment priorities in Italy
Source: Orphanet J Rare Dis. 2024 Jul 12;19:264. doi: 10.1186/s13023-024-03272-0 (PMC11241840; doi:10.1186/s13023-024-03272-0)
Supplement: Supplementary file 1 — Additional file 1. [file 13023_2024_3272_MOESM1_ESM.docx]

**Appendix A**

**FXS SECTION**

1. Which of the following best describes you?
   - __ I have Fragile X syndrome
   - __ I am a family member or caretaker of someone with FXS
   - __ I am a person with *FMR1* premutation
   - __ I am a parent with *FMR1* premutation
   - __ I am a professional who works with a person with FXS
2. What is the **age** of the **person with FXS** with whom you have the connection? Or your age if you have FXS:
   - __ Male: Birth to 5 years old
   - __ Female: Birth to 5 years old
   - __ Male: 6 to 12 years old
   - __ Female: 6 to 12 years old
   - __ Male: 13 to 21 years old
   - __ Female: 13 to 21 years old
   - __ Male: 22 years and older
   - __ Female: 22 years and older
3. Assign a score from 1 to 5 to the characteristics below based on how much you think they **impact on the life** of the person with FXS (1=most impact and 5=least impact):
   - __ Anxiety—anticipatory, e.g., of new/upcoming events
   - __ Auditory processing difficulties—being able to listen to instructions and react to them
   - __ Autism
   - __ Communication delays—initiation (asking for help) and social (turn-taking)
   - __ Hyperactivity
   - __ Learning or Intellectual disability (problems with abstract thinking, learning)
   - __ Memory—short term
   - __ Memory—long term
   - __ Motor delays (e.g., low muscle tone, poor fine motor skills, poor balance)
   - __ Motor stereotypes (hand-flapping, spinning around)
   - __Perseveration—speech (repeating things over and over)
   - __ Seizures
   - __ Sensory processing difficulties
   - __ Short attention span
   - __ Social anxiety
   - __ Speech/Language delays—receptive (understanding spoken language)
   - __ Speech/Language delays—expressive (speaking spoken language)
   - __ Visual information processing difficulties
   - __ Difficulties in planning and scheduling activities.
   - __Difficulties in autonomies in self-care and at home (washing, dressing, cooking)
   - __Difficulties in social-relational autonomies (organizing meetings and one's own commitments, respecting social rules, etc.).
   - __Difficulties in work and/or schooling
   - __ Other – Describe:____________________________________________________________
4. Assign each of the following areas a score from 1 to 3 based on the extent to which you think it affects the **daily life of the person with FXS** (1 = greatest impact; 3= least impact):
   - __ Behavior
   - __ Intelligence
   - __ Physical abilities
   - __ Language
5. Assign a score from 1 to 5 to the areas of daily life listed below based on how much you think they are **affected by in the person with FXS** (1=most affected and 5=least affected):
   - __ Ability to learn academic skills/reading/math
   - __ Ability to take care of self-care skills/hygiene/cooking
   - __ Ability to speak/communicate
   - __ Ability to be left alone/spend time alone
   - __ Ability to control behavioral outbursts
   - __ Ability to attend and perform at school
   - __ Ability to find/maintain job
   - __ Ability to make and maintain friends
   - __ Ability to live independently
   - __ Ability to establish and maintain a relationship
   - __ Ability to be like other people his/her age.
   - __ Ability to attend events where there are a lot of people/noise
   - __ Willingness to go to new places
   - __ Willingness to travel/ go on vacation
   - __ Other—Describe:_________________________________________________________________
6. Score each of the following aspects from 1 to 5 according to how **challenging** you think it is to live with FXS (1=most challenging and 5=least challenging):
   - __ Always thinking—how are things going, what do I need to do next? Needing to always be ‘one step’ ahead
   - __ Checking in/setting up daily programming—school, work, etc.
   - __ Doctor/dentist appointments—finding/attending
   - __ Doing activities with friends—both child’s and adult’s
   - __ Extra costs—therapies, medications, clothing, glasses, laundry
   - __ Extra time it takes to do everything
   - __ Finding respite
   - __ Food—Always hungry/wants to eat out
   - __ Handling behaviors (negative)—tantrums, aggression, spitting, cussing
   - __ Hygiene—shower, toileting, hair cuts
   - __ Impact on non-affected family members
   - __ Medications—getting prescriptions/not running out/ making changes
   - __ Need for constant supervision
   - __ Needing to make sure everything is “set” for the day—routine, visuals
   - __ Person doesn’t understand directions/can only do one thing at a time
   - __ Person is unable to tell you what he/she wants/needs
   - __ Running errands—how many stops can I make? What environments could be hard/noisy?
   - __ Sleeping
   - __ Worry about the future
   - __ Other—Describe
7. List three of your **favourite things** about the person with FXS. (Or three things you like about yourself).

__________________________________________________________________________________

1. What are the top three aspects of Fragile X syndrome that you would like to see a **treatment address**_**__________________________________________________________________________**

**PM SECTION**

**1.** What is your **gender**?

Male

Female

Don’t want to specify

2. What is your **age** or what is the age of the person with premutation with whom you have the connection?

0-5 years old

6-12 years old

13-21 years old

22-45 years old

46-65 years old

66 years and over

3. Assign a score from 1 to 5 to the characteristics below based on how much you think they im**pact on your life** (1=most impact and 5=least impact):

Anxiety—anticipatory, e.g., of new/upcoming events

Auditory processing difficulties—being able to listen to instructions and react to them

Autism

Communication delays—initiation (asking for help) and social (turn-taking)

Hyperactivity

Learning or Intellectual disability (problems with abstract thinking, learning)

Short-term memory

Long-term memory

Delayed motor development (e.g., low muscle tone, poor fine-motor skills, poor balance)

Motor stereotypies (e.g., hand or head flapping)

Perseveration and repetitiveness in speech (repeating things over and over again)

Seizures

Difficulty with sensory processing

Reduced attention span

Social anxiety

Language delays (comprehension of spoken language).

Language/expressive delays (production of spoken language).

Disorders in language pragmatics (difficulty in understanding others' intentions, difficulty in understanding abstract language, etc.

Difficulty in processing visual information.

Difficulties in planning and scheduling activities.

Difficulties in autonomies in self-care and at home (washing, dressing, cooking)

Difficulties in social-relational autonomies (organizing meetings and one's own commitments, respecting social rules, etc.).

Difficulties in work and/or schooling . .

Other: Describe

4. Assign a score from 1 to 5 to the **areas of daily life** listed below based on how much you think they affect your life (1=most affected and 5=least affected).

Ability to learn academic skills/reading/math

Ability to take care of self-care skills/hygiene/cooking

Ability to speak/communicate

Ability to be left alone/spend time alone

Ability to control behavioral outbursts

Ability to attend and perform at school

Ability to find/maintain job

Ability to make and maintain friends

Ability to live independently

Ability to establish and maintain a relationship

Ability to be like other people his/her age.

Ability to attend events where there are a lot of people/noise

Willingness to go to new places

Willingness to travel/ go on vacation

Other: Describe

5. Which five specific aspects of daily living with premutation are the **most challenging**? Prioritize 1, 2, 3, 4, 5.

Always thinking—how are things going, what do I need to do next? Needing to always be ‘one step’ ahead

Checking in/setting up daily programming—school, work, etc.

Doctor/dentist appointments—finding/attending

Doing activities with friends—both child’s and adult’s

Extra costs—therapies, medications, clothing, glasses, laundry

Extra time it takes to do everything

Finding respite

Food—Always hungry/wants to eat out

Handling behaviors (negative)—tantrums, aggression, spitting, cussing

Hygiene—shower, toileting, hair cuts

Impact on non-affected family members

Medications—getting prescriptions/not running out/ making changes

Need for constant supervision

Needing to make sure everything is “set” for the day—routine, visuals

I don’t or person doesn’t understand directions/can only do one thing at a time

I am or person is unable to tell you what he/she wants/needs

Running errands—how many stops can I make? What environments could be hard/noisy?

Sleeping

Worry about the future Other: Describe:

1. Check if you exhibit:

Fragile X -associated tremor ataxia syndrome (FXTAS)

Fragile X-associated primary ovarian insufficiency (FXPOI)

Fragile X -associated neuropsychiatric disorders (FXAND)

Other: Specify:

1. What are the **top three aspects** of *FMR1* premutation that you would like to see a

**Treatment address**.
